# Supplementary material for: Quality of life among leprosy patients: An analysis of associated factors
Source: PLOS Ment Health. 2025 May 30;2(5):e0000329. doi: 10.1371/journal.pmen.0000329 (PMC12798199; doi:10.1371/journal.pmen.0000329)
Supplement: Attachment — Submitted filename: Response Reviewers.pdf [file pmen.0000329.s002.pdf]

Dear Kizito Omona, PhD,

Academic Editor, PLOS Mental Health

Dear Editor, this is regarding the manuscript **PMEN-D-24-00585** entitled as "**Quality of Life among Leprosy Patients: An Analysis of associated Factors**" submitted to PLOS Mental Health. Thank you for the opportunity to respond to the constructive reviews provided to our submission to PLOS One. We are grateful that reviewers 1, 2 and 3 agree that this is a technically sound piece of research and hope we have satisfied both reviewers concerns about the rigor of the statistical assessment. We appreciate that all reviewers agree we have been transparent with provision of the data on which our conclusions are based and have presented the results in intelligible language. We have addressed all reviewer comments in the revised manuscript now respectfully submitted as both marked (Revised Manuscript with Track Changes) and unmarked (Manuscript) versions.

We have made every effort to comply with the PLOS Mental Health Style requirements in accord with journal templates. Before addressing the specific reviewer comments point-by-point below, we would like to respond to your editorial request as follow:-

Journal Requirements:

1. We suggest you thoroughly copyedit your manuscript for language usage, spelling, and grammar. If you do not know anyone who can help you do this, you may wish to consider employing a professional scientific editing service.

Response: Dear academic editor, we appreciate your valuable input. We have revised the manuscript to address the grammatical errors by refining the spelling, diction, and syntax. We are confident that the updated version now offers improved clarity and readability for our readers.

Whilst you may use any professional scientific editing service of your choice, PLOS has partnered with both American Journal Experts (AJE) and Editage to provide discounted services to PLOS authors. Both organizations have experience helping authors meet PLOS guidelines and can provide language editing, translation, manuscript formatting, and figure formatting to ensure your manuscript meets our submission guidelines. To take advantage of our partnership with AJE, visit the AJE website (http://learn.aje.com/plos/) for a 15% discount off AJE services. To take

advantage of our partnership with Editage, visit the Editage website (www.editage.com) and enter referral code PLOSEDIT for a 15% discount off Editage services. If the PLOS editorial team finds any language issues in text that either AJE or Editage has edited, the service provider will re-edit the text for free.

Upon resubmission, please provide the following:

- The name of the colleague or the details of the professional service that edited your manuscript

- A copy of your manuscript showing your changes by either highlighting them or using track changes (uploaded as a *supporting information* file)

- A clean copy of the edited manuscript (uploaded as the new *manuscript* file)

Additional Editor Comments (if provided):

Address the concerns raised by the reviewers

Response: Dear academic editor, Thank you so much for your suggestion.

Review Comments to the Author

Please use the space provided to explain your answers to the questions above. You may also include additional comments for the author, including concerns about dual publication, research ethics, or publication ethics. (Please upload your review as an attachment if it exceeds 20,000 characters)

Reviewer #1: I am pleased to write this recommendation regarding their recent publication .The work presented is not only thorough and well-researched but also offers valuable insights into the field of mental health and chronic infectious disorders. Throughout the paper, the authors demonstrates a strong command of the subject matter, backed by a solid methodology and clear presentation.

Response: Dear academic reviewer, we sincerely appreciate your endorsement and the timely publication suggestion. Thank you for your valuable feedback.

Reviewer #2: Abstract:

First sentence in line 21 doesn’t add any significance information to the abstract.

Response: Dear reviewer, Thank you so much for your suggestion. We have carefully considered and it has been done in the revised version.

Same applies to lines 26 and 27: "Consequently, there is limited information available on26 the quality of life of patients with leprosy in Ethiopia, particularly in Eastern Ethiopia."

Response: Dear reviewer, Thank you for your feedback. We have thoroughly reviewed and incorporated your suggestions into the revised version.

In lines 28 to 28, the objective of the study does not match the literature gap (mental health) highlighted above.

Response: Dear reviewer, Thank you for your thoughtful input. The main objective of this study was to assess the quality of life and its associated factors among leprosy patients in Eastern Ethiopia. As such, the assessment encompassed the physical, psychological, social, and environmental domains of quality of life, with a specific focus on mental health aspects.

From lines 32 to 37, the authors provided excess explanations that are not required in abstract. Instead mention the key activities. for instance, consider deleting information on "completeness and consistency", reasons for using linear regression ...

Response: Thank you very much for your suggestion. We have removed the suggested information from the revised manuscript.

Introduction

Lines 74 and 75: Two consecutive sentences started with the word "despite". Consider revising it.

Be consistent with the use of abbreviations for Quality of Life.

Response: Dear reviewer, Thank you for your feedback. We have incorporated your suggestions into the revised version.

Choose between QOL and QoL.

Response: Dear reviewer, Thank you for your feedback. QOL is replaced with QoL the revised version.

Lines 84 to 96, the ideas are scattered. Authors should organize their presentation well. for instance, they may start a description of the psychoclogical repercussion, then the causes (clinical or environmental) and manifestation in the lifes of the patients, etc....

Response: Dear reviewer, Thank you for your feedback. We have modified the revised version.

Line 103: check grammatical error "which resulting..."

Response: Dear reviewer, Thank you so much for your suggestion. We have carefully considered and it has been done in the revised version.

Line 104-105: "effects of stigma", has seem to be repeated in the Intro. Unless the authors intends to provide statistical evidence, they should consider reorganizing the flow of the writeup well.

Response: Dear reviewer, Thank you so much for your suggestion. We have modified it in the revised version.

Although the authors have provided some empirical evidence on mental health and leprosy, a more elaborate review is needed to determine the literature gap or context gap that this study is filling. Otherwise they should provide a stronger justification for the study.

Response: Dear reviewer, Thank you for your feedback. We have incorporated your suggestion into the revised version. Your input is greatly appreciated.

Methods

Line 133: Authors should explain the research design in the context of the study and give justifications. They should be mindful not to just give generic definitions from literature.

Response: Dear reviewer, Thank you for your valuable input. In response to your feedback, we have conducted an institutional-based cross-sectional study design for this research. This design was chosen based on its appropriateness and alignment with the study's objectives and context.

Line 134: Further description of the study population should be considered indicating eligibility criteria. Authors should explain why other skin diseases were considered and include this information in the background. If the study is not just about leprosy, then the authors may consider revising the topic as well as the objective of the research.

Response: Dear reviewer, Thank you for bringing up this point. We have addressed the study population and eligibility criteria to provide clarity on the inclusion and exclusion criteria.

Line 142: What is the eligibility criteria?

Response: Dear reviewer, Thank you for bringing up this point. Eligibility criteria were patients with leprosy whose age is ≥18. Patients diagnosed with leprosy who presented with concurrent skin diseases, or physical disabilities unrelated to leprosy were excluded from the study.

Line 138 to 143: How did the authors calculate the sample size?

Response: Dear reviewer, Thank you for raising this important point. We have provided detailed information on how the sample size was determined in the manuscript.

Lines 149 to 159: What is the reliability of the WHOQOL-100 tool?

Response: Dear reviewer, Thank you so much for your concerns. WHOQOL-100 tool was developed by WHO and has shown high internal consistency, based Cronbach's alpha values.

Lines 161 to 172: HQ9, GAD7, ASSIST, EMIS-AP and SBQ-R were not mentioned in the Abstract. Authors should consider correcting this omission.

Response: Dear reviewer, Thank you so much for your concerns.

Discussion

Well written discussion. Authors should consider referencing specific results in the discussion of the various themes. But be cautious not to present too many results which could result in repetition of the results section.

Response: Dear academic editor, Thank you so much for your suggestion. We have carefully considered and it has been done in the revised version.

Reviewer #3: Overall an interesting and important article. However, the manuscript is difficult to read and understand in large part due to a multitude of grammatical errors and awkward phrasing of sentences. Please consider the suggestions below to improve the quality of the paper and make it more suitable for publication.

Response: Dear reviewer, we appreciate your valuable input. We have revised the manuscript to address the grammatical errors by refining the spelling, diction, and syntax. We are confident that the updated version now offers improved clarity and readability for our readers.

- Line 76, insert comma between “curable” and “leprosy”

Response: Dear academic editor, Thank you so much for your suggestion.

- Line 76, insert “is” between “leprosy” and “associated

Response: Dear reviewer, Thank you so much for your suggestion. It has been done in the revised version

- Line 76, change the “e” in discrimination to an “a”

Response: Dear reviewer, Thank you so much for your suggestion. It has been modified in the revised version.

- Lines 77-78, the word affected is used consecutively in the same sentence. Consider the following rephrase instead: the psychological health of patients living with leprosy is affected by several economic and social factors intimately connected with QoL.

Response: Dear reviewer, Thank you so much for your suggestion. It has been done in the revised version

- Regarding the introduction, for better flow of the paper, recommend describing “quality of life” prior to discussing how QoL is decreased in patients living with leprosy (i.e. place lines 79-83, before lines 74-78). This will better contextualize the argument of the paper for your readers.

Response: Dear reviewer, Thank you for your insightful recommendation regarding the flow of the paper. We have incorporated your suggestion in the revised version to enhance the contextualization of the argument for our readers.

- Line 85, it should say “patients” not “patient”; throughout the manuscript, the authors tend to say “patient” where “patients” instead should be used, please make this correction throughout the manuscript.

Response: Dear reviewer, Thank you so much for your suggestion. It has been done in the revised version

- Line 88, discuss further what these misconceptions are or provide examples of the most common misconceptions held about leprosy

Response: Dear reviewer, Thank you for your valuable suggestion. Some of the misconceptions surrounding leprosy, were such as the belief that it is highly contagious and can be easily transmitted through casual contact, as well as the misconception that leprosy is a curse or divine punishment.

- Recommend removing lines 88-89 or condensing with the earlier sentences (lines 86-88) as you are now repeating the same points without expanding further on them

Response: Dear reviewer, Thank you so much for your suggestion. It has been done in the revised version

- Line 89, patient should be the plural for, patients

Response: Dear reviewer, Thank you so much for your suggestion. It has been done in the revised version

- Recommend rephrasing lines 93-85. For example: "The social lives of patients with leprosy are often non-existent, rather these patients often endure isolation, primarily stemming…"

Response: Dear reviewer, Thank you so much for your suggestion. It has been done in the revised version

- Lines 97-98, consider the following rephrase: "patient with leprosy are 30% more likely to experience mental disorders globally, of which 70% experience depressive disorders while another 28% experience anxiety disorders"

Response: Dear reviewer, Thank you so much for your suggestion. It has been modified in the revised version

- Line 100-102, this sentence as it is currently written is very confusing, consider rewriting entirely: Studies conducted in Nigeria have shown that of patients with leprosy, 14% have depression while 34.6% have anxiety.

Response: Dear reviewer, Thank you so much for your suggestion. It has been modified in the revised version

- Line 103, change “leprosy of patients” to “patients with leprosy”

Response: Dear reviewer, Thank you so much for your suggestion. It has been changed in the revised version

- Lines 103-104, the sentence that comes after “leprosy of patients” does not immediately make sense, what is the connection between this sentence and the one before it (lines 102-103), what is the conclusion/point being made here? Please rephrase for better clarification

Response: Dear reviewer, Thank you so much for your suggestion. It has been modified in the revised version

- Line 113, insert a period between society and furthermore, as furthermore should be the start of a new sentence

Response: Dear reviewer, Thank you so much for your suggestion. It has been modified in the revised version

- Line 128, change “is” to “was”

Response: Dear reviewer, Thank you so much for your suggestion. It has been changed in the revised version

- Lines 129-130, the phrasing of this sentence is confusing, please rephrase for clarity

Response: Dear reviewer, Thank you so much for your suggestion. It has been modified in the revised version

- Line 130, what does OPD stand for? Since this is the first mention of the acronym, the full name should be written out for readers

Response: Dear reviewer, Thank you so much for your suggestion. (OPD) stands for outpatients department.

- Please explain the study design further. When institutions were apart of the project? Was there an IRB protocol that was approved by all institutions? This section should provide more detail than what is current presented in the manuscript

Response: Dear reviewer, Thank you for your inquiry regarding the study design and ethical considerations. The research was conducted using a cross-sectional study design. Ethical approval for the study was obtained from the Institutional Health Research Ethics Review Committee (IHRERC) at the College of Health and Medical Sciences of Haramaya University, with reference number Refw-IHRERC/141/2024

- Line 134, in the population section, please describe your inclusion and exclusion criteria further. Were all patients with leprosy accepted for the study regardless of age? Why were those with leprosy but other skin disease excluded from consideration?

Response: Dear reviewer, Thank you for bringing up this point. Eligibility criteria were patients with leprosy whose age is ≥18. Patients diagnosed with leprosy who presented with concurrent skin diseases, or physical disabilities unrelated to leprosy were excluded from the study.

- Line 155, this phrase is confusing and not needed “which agreed as a five-point liker scale?” Please either rephrase the sentence or remove

Response: Dear reviewer, Thank you so much for your suggestion. It has been changed in the revised version.

- Lines 175-176, when defining “ever substance use” and “current substance use,” please clarify if these are the definitions that the authors themselves came up with for the purposes of the study or if they are the definitions associated with the ASSIST screen

Response: Dear reviewer, Thank you so much for your suggestion. Definitions associated with the ASSIST screen.

- Line 179, change EMIC-AP to EMIS-AP

Response: Dear reviewer, Thank you so much for your suggestion. It has been changed in the revised version.

- Line 194, consider this rephrase instead: “one supervisor who oversaw the data collection process”

Response: Dear reviewer, Thank you so much for your suggestion. We accept and it has been done in the revised version.

- Line 197-202, what were the convenient locations? How long were the interviews held for/on average how long interviews were?

Response: Dear reviewer, Thank you so much for your suggestion.

- Lines 203-211, it is not necessary to write out the dependent and independent variables in this format. They should just be included in the tables and charts associated with the manuscript

Response: Dear reviewer, Thank you so much for your suggestion. This is only overview of the study variables that the readers can easily understand.

- Line 233, there should be a comma between quality and pre-test

Response: Dear reviewer, Thank you so much for your suggestion. We accept and it has been done in the revised version.

- Line 268, what constitutes poor social support? How was this being defined for participants? How did participants define their social support as poor?

Response: Dear reviewer, Thank you so much for your suggestion. In this study, social support was operationalized as**:** Individual who score on OSS-3: 12 to 14 indicate Strong social support, 9 to 11 indicate Intermediate social support and 3 to 8 indicate Poor social support (Dalgard et al., 1995)

We reaffirm our dedication to upholding rigorous research standards and academic excellence. The revisions made to our manuscript have taken into account all the feedback provided by the reviewers and align with the guidelines set forth by the journal. We eagerly anticipate your favorable evaluation of the revised manuscript for publication in the near future. Thank you for your consideration.

Sincerely,

Alo Edin (Corresponding author on behalf all authors)
